# Pathogenic *Neurofibromatosis type 1* gene variants in tumors of non‐NF1 patients and role of R1276

**DOI:** 10.1002/2211-5463.70157

**Published:** 2025-11-11

**Authors:** Mareike Selig, Swanhild Lohse, Sara Elahi, Nils Hartmann, Stefanie Deckert, Shutian Si, Alexander Desuki, Anja Harder

**Affiliations:** ^1^ Institute of Medical Genetics University Medical Centre Johannes Gutenberg University (UMC) Mainz Germany; ^2^ CURE‐NF Research Group, Medical Faculty Martin Luther University Halle‐Wittenberg Germany; ^3^ Institute of Pathology University Medical Center Johannes Gutenberg University (UMC) Mainz Germany; ^4^ Max‐Planck Institute for Polymer Research Mainz Germany; ^5^ University Cancer Centre (UCT) Mainz, University Medical Centre Johannes Gutenberg University (UMC) Mainz Germany; ^6^ Medical Faculty University of Muenster Germany

**Keywords:** mutation, Neurofibromatosis type 1, somatic, tumor, variant

## Abstract

Neurofibromatosis type 1 (NF1) is a tumor predisposition syndrome associated with pathogenic variants affecting the GTPase‐activating protein neurofibromin. Genetic variants affect neurofibromin through targeted protein degradation, failed aggregation of the monomers or failure of specific domains depending on the functional state. In addition to the occurrence in NF1, there is evidence of pathogenic variants occurring in various solid tumors. We collected data from 63 patients from our molecular tumor board for *NF1* gene sequencing and detected 72 *NF1* variants, thereby 32% of those being pathogenic. They occurred most often in lung cancer, glioma, melanoma, sarcoma, and gynecological cancer and affected women more often. Pathogenic *NF1* variants appeared at low frequency except in malignant melanoma and glioma (10%). We present common pathogenic variants, their types, and association with tumor entities, their frequency, and domain localization and focus on common recurrent variants and their probable result and predictive quality in somatic mutation screening. We detected variants in different tumor entities without NF disease, covering more frequent truncating mutations than reported for germline. We question whether all *NF1* variants reported in tumors without the presence of NF1 are somatic. To conclude, recognition of *NF1* mosaicism requires multitissue sampling, precise sequencing technologies, and inclusion of genetic counseling.

## Graphical abstract

Somatic variants of the neurofibromatosis type 1 (*NF1*) gene occur across neoplasms without clinical manifestation of the disease NF1. We identified emerging somatic pathogenic *NF1* variants and hotspots, for example, at the arginine finger 1276. Those missense variants provide fundamental information about neurofibromin's role in cancer. Somatic *NF1* mosaicism is more common than assumed, demanding greater attention in tumor boards.

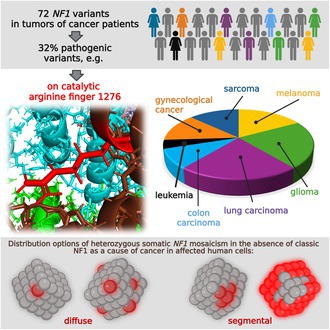

**Abbreviations**
ACMG: American College of Medical Genetics and Genomics
AF: Allele frequency
FFPE: formalin fixed paraffin embedded
GAP: GTPase‐activating protein
GRD: GTPase‐related domain
MPNST: malignant peripheral nerve sheath tumors
MTB: molecular tumor board
NF: Neurofibromatosis
NF1: Neurofibromatosis type 1
NGS: Next‐generation sequencing
RAS: Rat Sarcoma viral oncogene

Neurofibromatosis type 1 (*NF1*, OMIM 16220) is an autosomal dominant tumor predisposition syndrome affecting all cells of the body. Clinical diagnostic criteria were updated in 2021 [1] and include café‐au‐lait macules, freckling, neurofibromas, Lisch nodules, optic pathway gliomas, and osseous lesions. Pathogenic germline variants of the highly conserved *NF1* gene (Neurofibromin, OMIM *613113, 17q11.2) are causative. They distribute across the entire coding and noncoding region or can occur as larger deletions and microdeletions. They reduce the expression, stability, and activity of neurofibromin in all haplo‐insufficient cells of the body (*NF1* ^+/−^). Neurofibromin is a GTPase‐activating protein (GAP) and acts as a tumor suppressor by negatively regulating Ras. The protein is composed of several domains and acts either in an auto‐inhibited or open functional state [2]. Pathogenic variants can thereby also induce targeted protein degradation via a destabilization of the wild‐type neurofibromin by dominant‐negative effects [3]. In a recent study tetramerization of neurofibromin was reported clearly complicating the prediction of mutations as the structure interfaces might be buried or exposed according to monomerization, dimerization, or tetramerization [4].

A failed creation of the correct neurofibromin protein structure (dimerization/tetramerization) may explain a general increase in Ras signaling in NF1‐associated cells, even if the central GTPase‐related domain (GRD) of neurofibromin is not altered. Failure of the interface between Ras and the GRD explains the high impact of missense mutations such as R1276P on malignant tumor growth in NF1 individuals early in disease development [5]. Figure 1 demonstrates that a mutational effect depends on both localization and conformation including the specific role of the affected residue. One of the utmost best‐studied *NF1* mutations of the arginine finger R1276 is affecting the GRD in neurofibromin, which is crucial for binding to Ras proteins and accelerating the hydrolysis of GTP to GDP in Ras. R1276 will convey its effect in dependence on the open and occluded formation within the dimer widening the quantity of options for mutational effects at this site. As shown for the neurofibromin tetramer, the arginine finger is always exposed within this structure thus reducing the possibilities for mutations at this site to a manageable size. Other sites when buried in the tetramer structure are subjected to a completely different prediction. In the case of nonsense mutations, remaining domains of neurofibromin might still interact with binding partners to facilitate some of the cellular functions. Future knowledge of detailed neurofibromin functions will elucidate the effects of pathogenic variants more precisely but will certainly demonstrate also a more diverse range of mutational effects.

**Fig. 1:**
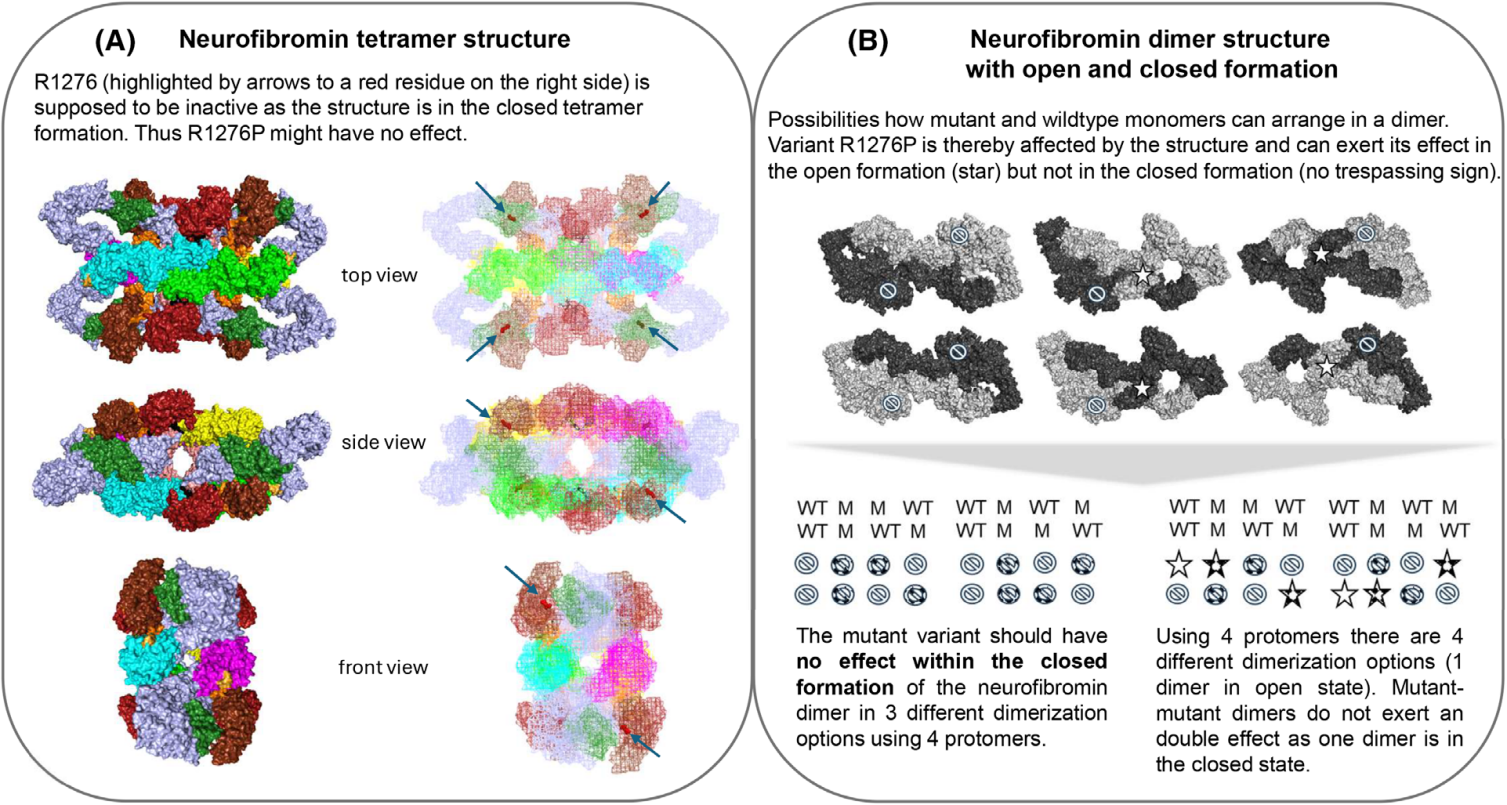
Effects of mutations using the example of the arginine finger depending on the protein structure. (A) The residue R1276 is highlighted within the novel neurofibromin tetramer structure. Surface view of the tetramer structure of NF1 isoform I (2,818 aa) with main domains labeled as following: N‐HEAT (light blue 1‐1189aa; (14)), Linker 1, 2 and 3 (black; (14)), CSRD (green forest 543–909 aa; Mo et al. 2022), TBD (salmon, 1095‐1197aa; Mo *et al*. 2022), GRD (orange, 1197–1525 aa; (14)), RasGAP (chocolate 1209–1463 aa; Rabara *et al*. 2019), Sec14‐PH (1545–1814 aa; 14), C‐HEAT (protomer 1—green, protomer 2—cyan, protomer 3—magenta, protomer 4—yellow (1830–2818 aa; (14)); S‐rich domain (bright orange 2432–2589 aa; (14)). (B) The residue is also demonstrated in the dimer using the PDB Model by Nashberger et al (14) with one protomer colored in light gray and one in dark black (PyMOL by Schrödinger 3.1.0). On the right, the occluded (inactive) neurofibromin is shown, whereas in the middle and on the left, variants of the open (active) form are demonstrated. A formation with both protomers open was not yet identified. Approximate localization of residue 1276 is marked by a star to indicate open/active GRD and with a no trespassing sign to indicate closed/inactive state of GRD. The different possibilities arising from dimer formations are shown below the gray arrowhead and illustrate the result on the mutational effect. White symbols indicate wild‐type protomers, dotted show R1276P mutant protomers. This image does only apply to pathogenic variants that do not affect the structure and refer to current neurofibromin model. It therefore can be surmised that mutational effects are very wide according to localization, structure and function.

NF1 patients face an increased risk of brain tumors, malignant peripheral nerve sheath tumors (MPNST), breast cancer, and other malignancies [6, 7]. Compared to the normal population, several cancers are more common in NF1 (gliomas, sarcomas, breast cancer, endocrine cancers, melanomas, acute lymphoblastic leukemias, ovarian and prostate cancer, and meningioma), and some occur at an earlier age (MPNST, breast cancer, and glioma) or even appear to be more deadly in NF1 patients (undifferentiated pleomorphic sarcomas, high‐grade gliomas, MPNST, ovarian cancer, melanoma; https://www.cancer.gov/news‐events/cancer‐currents‐blog/2021/nf1‐associated‐with‐more‐cancer‐types). Irrespective of the presence of the disease NF1, however, there is increasing evidence that pathogenic variants of the *NF1* gene occur in various solid tumors [8, 9]. This underlines the extending role of neurofibromin beyond the tumor syndrome. Functional loss of neurofibromin can cause resistance to kinase inhibitors and is therefore important for therapy selection underlining the importance of pre‐therapeutic *NF1* testing such as genotype‐to‐drug resistance phenotype maps of *NF1* variants [10, 11].

Nevertheless, many questions remain unanswered: Is there a correlation between *NF1* variants and tumor types? Are there mutation hotspots for tumor entities that are relevant for testing in a clinical setting? Are these variants truly somatic or can they also be mosaic germline or late postmitotic mosaic variants that only occur in a specific region of the body as in segmental NF1 or even only in a single tissue? If so, a mosaic variant would not lead to a clinically fully developed NF1, but to a subclinical manifestation. However, it would be associated with an increased tumor risk in the affected cells or body regions. Even if a genetic screen in tumors is ruling out the presence of the same variant in blood, we hypothesize that mosaicism can be overlooked. This study aimed to determine the prevalence and spectrum of pathogenic *NF1* variants in various tumor entities and assess their impact. A more detailed *NF1* variant analysis is important to achieve more precise individualized patient care in oncology.

## Materials and methods

In the Mainz molecular tumor board (MTB), the following inclusion criteria had to be met for molecular genetic testing to take place: Age ≤ 50 years and/or rare entity and/or advanced tumor disease and/or aggressive disease course and/or tumor progression after guideline‐based therapies. The patients discussed in MTB have provided written informed consent allowing their data to be analyzed in scientific projects.

The study was reported to and approved by the local ethics committee of the state chamber of physicians (Landesärztekammer) of Rhineland‐Palatinate (no. 2024‐17489). This research complies with the guidelines for human studies and was conducted ethically in accordance with the World Medical Association Declaration of Helsinki.

We collected data from patients (*n* = 63; female: 33, male: 30, mean age: 61,2) reviewed in our MTB at the University Mainz Oncology Centre who provided tumor material for next‐generation sequencing (NGS) of the entire coding sequence of the *NF1* gene. We analyzed 72 *NF1* variants in these 63 patients from 1 March 2022 to 31 October 2024 (during this period we investigated 1279 cases in 1078 patients at all in the molecular tumor board). The tumor material was formalin‐fixed paraffin‐embedded (FFPE) tissue, whereby the tumor area was evaluated and marked by a pathologist prior to processing. The tissue was scraped off and subjected to DNA and RNA preparation. After quality analysis, a library was created using the QIAseq Multimodal Pan‐Cancer Panel (Qiagen, Hilden, Germany). Sequencing was performed with the NextSeq 500 or NexSeq 2000 platforms (Illumina, San Diego, CA, USA) using 2 × 150 bp reads and a custom sequencing primer for read 1 (QIAseq A Read1 Primer I). Generated FASTQ files were analyzed using several software tools provided by the CLC Genomics Workbench (Qiagen). Sequencing reads were aligned to the human reference genome GRCh37 (hg19) and identified variants were based on RefSeq accession numbers. Polymorphisms were excluded by filtering against dbSNP and the detection limit of variants was set to 5% allele frequency. Furthermore, only targets with a minimum coverage above 50 reads were considered for further analysis. For data analysis, the software CLC Genomics Workbench (Qiagen) was applied. Variants were further annotated using the following databases and tools: VarSome (https://varsome.com/), cBioPortal (https://www.cbioportal.org) and ClinVar (https://www.ncbi.nlm.nih.gov/clinvar/). We classified variants into benign, likely benign, variants of uncertain significance, likely pathogenic or pathogenic according to ACMG Guidelines approved standard [12] and recognized the somatic classification tiers [13]. According to our established reporting system, *NF1* gene variants classified as polymorphism or as benign or probably benign using ClinVar as well as variants with a pathogenicity score ≤ 0 were neither assessed nor reported. The classification of *NF1* variants as somatic or germline variants was based on family and clinical history, clinical presentation (presence or absence of syndromic signs of NF1) and comparison with germline test results, if available. We mapped all *NF1* variants in malignant tumors, both for our institutional cohort and using database resources. We compared variants from our cohort with different trial cohorts listed at the open resource cBioPortal for Cancer Genomics (https://www.cbioportal.org, latest query: 24 September 2024 with querying 10 967 samples/10 953 patients in 32 studies). To design heat and Jaccard maps to demonstrate pairwise overlap of studies, we established a matrix from published variants of cited studies and of our cohort, calculated the Jaccard index and visualized data using the latest Python package (released: 31 July 2025) matplotlib 3.10.5 PyPl (https://pypi.org/project/matplotlib/).

## Results

### Overview of *NF1* variants of the molecular tumor board at Mainz’ oncology center

**Fig. 2:**
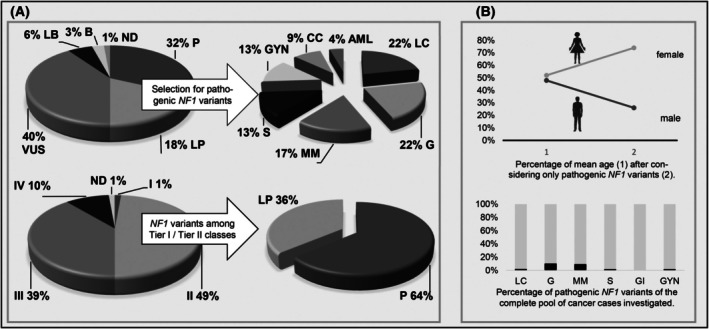
Detection of *NF1* variants in various tumor entities investigated by *NF1* gene sequence analysis and in the absence of the disease NF1. (A) Overview of variants reported in‐house (B, benign; I, Tier I;II, Tier II; III, Tier III; IV, Tier IV; LB, likely benign; LP, likely pathogenic; ND, not determinedP, pathogenic; VUS, variant of unknown significance). Tumor types among pathogenic variants are displayed (AML, acute myeloid leukemia; CC, colorectal cancer; G, glioma; GYN, gynecological cancer; LC, lung cancer; MM, malignant melanoma; S, sarcoma) right hand. (B) Distribution of age and sex are shown among all pathogenic *NF1* variants.

We evaluated data of a total of 72 *NF1* variants in 63 patients of the Mainz patient pool, and detected those in various tumor entities not associated with the disease NF1 (Table S1 & Fig. S1). Half of all variants were assigned to Tier I (1%) and II (49%), whereas Tier III also made up a significant feature (Fig. 2). Nevertheless, to current knowledge, only Tier I and Tier II variants are used for strategic decisions in oncology, which is reducing the frequency of relevant *NF1* variants to half in the Mainz cohort. As special attention should be given to the pathogenic variants, we assessed all variants in terms of their pathogenicity: Only 32% turned out to be pathogenic. Many of the variants represented likely pathogenic variants (18%) or variants of uncertain significance (40%). Taking it strictly, we focused on the pathogenic variants to determine the influence on tumor formation with absolute certainty. Correcting this measure in our cohort, pathogenic *NF1* variants were most often found in lung cancer, glioma, malignant melanoma, sarcoma, and gynecological cancer and affected many more women than men (74 versus 26) (Fig. 2): In contrast, data reported at cBioPortal more frequently cover gynecological cancer (Table S1 & Fig. S1). All pathogenic *NF1* variants are shown in Table 1. We then considered the real frequency of pathogenic *NF1* variants within the pool of all patients investigated in our time frame. As shown (Fig. 2B), *NF1* variants occur still at a low frequency except in malignant melanoma and glioma.

**Table 1:** Pathogenic *NF1* variants of tumor patients at the Mainz Oncology center from 1 March 2022 to 31 October 2024. Pathogenic *NF1* variants that have been detected in different tumor entities (AML, acute myeloid leukemia; CC, colon cancer (all types); G, glioma (all grades); GYN, gynecological cancer (all types including breast cancer); LC, lung cancer (all types); MM, malignant melanoma; S, sarcoma (including MPNST)) with respect to mutation type and to reported frequencies > 10% at cBioPortal which is the largest collection so far. Variants are allocated to domains of neurofibromin: N‐HEAT (N‐terminal Huntington, Elongation Factor 3, PR65/A, TOR; AA 1–1189; [31]), CSRD (N‐terminal cysteine‐serine‐rich domain; 543–909; [32]), TBD (tubulin binding domain, AA 1095–1197; [32]), GAP‐related domain, GRD (AA 1197–1546; [31]), central RasGAP domain (AA 1209–1484; [22]), C‐HEAT (C‐terminal Huntington, Elongation Factor 3, PR65/A, TOR; AA 1859–2839, [31]). Recurrent Mainz’ variants are highlighted in dark gray. Recurrent variants of the cBioPortal are highlighted in light gray. Variant p.Q519 is additionally marked with ‘#’ and the variant at R1276 is additionally marked with ‘##’ as they are reported in other than cBioPortal datasets (15–17).

Neurofibromin domain	Pathogenic variant according to HGVS (recurrent variants marked)	Tumor entity	Mutation type	High frequency at cBioPortal
N‐HEAT	c.244_247del; p.Q83*	G	Nonsense	No
	c.487G>T; p.E163*	GYN	Nonsense	No
	c.1062G>A; p.K354=	MM	FS	No
	c.1555C>T; p.Q519*	MM	Nonsense	No **#**
N‐HEAT, CSRD	c.2033dup; p.I679Dfs*21	S	FS dup	No
	c.2033dup, p.I679Dfs*21	AML	FS dup	No
	c.2252‐1G>T	CC	Splice	No
N‐HEAT	c.2851‐1G>T	LC	Splice	No
N‐HEAT, TBD	c.3089C>G; p.S1030*	GYN	Nonsense	No
	c.3158C>G; p.S1053*	GYN	Nonsense	No
	c.3158C>G; p.S1053*	LC	Nonsense	No
GRD RasGAP	c.3658G>T; E1220*	LC	Nonsense	No
	c.3827G>A; R1276Q	G	Missense	Yes **##**
	c.4078C>T; p.Q1360*	MM	Nonsense	No
	c.4084C>T; p.R1362*	S	Nonsense	Yes
	c.4183C>T; p.Q1395*	LC	Nonsense	No
	c.4341G>C; p.Q1447H	S	Missense	No
C‐HEAT	c.5986del; p.I1996Yfs*16	CC	FS del	No
	c.6652del; p.R2218Efs*15	LC	FS del	No
	c.7037_7040del; p.D2346Vfs*49	G	FS	No
	c.7037_7040del; p.D2346Vfs*49	G	FS del	No
	c.7348C>T; p.R2450*	MM	Nonsense	Yes
	c.8155del; p.S2719QFS*20	G	FS	No

The allele frequency (AF) of pathogenic *NF1* variants in our cohort ranged from 10.5% to 94.3% (median 40%). Variants with an AF < 10% were excluded due to the challenge to distinguish them from artifacts. A clear correlation between specific variant types and corresponding AF thresholds was not observed. Detailed AF values for each variant are listed in Table S1.

### Spectrum of pathogenic *NF1* variants and affected tumor types

In our cohort, nonsense variants were most common (48%) followed by deletions/duplications (17%), missense variants (13%), frameshift variants (13%), and splicing errors (9%). In our patient pool, tumor entities with *NF1* variant frequency of > 10% included lung cancer, gliomas, melanomas, sarcomas, and gynecological tumors. Some tumor entities, which have been frequently reported in the past, are not represented at all in our Mainz oncology patient pool. Among pathogenic variants (summarized in Table 1), some missense variants (including silent base changes) were detected in our analysis: c.1062G>A/p.K354 = (N‐HEAT domain), c.3827G>A/R1276Q and c.4341G>C/p.Q1447H (GRD).

### Comparison of pathogenic *NF1* variants and affected tumor types to database entries

To contextualize our findings with the largest available pan‐tumor cohort, we evaluated reported data on cBioPortal and extracted 903 *NF1* mutations and 48 structural variants in 32 cancer types. The alteration frequency in the tumor tissue of > 10% was detected in cutaneous melanoma, endometrial carcinoma, lung squamous cell carcinoma, lung adenocarcinoma, bladder urothelial carcinoma, pheochromocytoma, and paraganglioma as well as ovarian serous cystadenocarcinoma. Overall mutation frequency was 6%. The distribution of variants continuously covered the whole gene with only some minor recurrent hot spots (in ≥ 5% of 10 953 patients truncating *NF1* variants R2540*/Q, R1362*, R440*/Q/P, R1306Q/*, and I697Dfs*21/P678Rfs10 as well as missense *NF1* variants R1870Q/X1870_splice and R1276Q were listed). Variant R2540* was specifically often reported in endometrial cancer reaching 10% frequency (most frequent *NF1* alterations in this uterine tumor type were R2450* (10/142), R1362* (6/142), X574_splice (3/142), and 1583* (3/142), but also reported in glioma and colon carcinoma). Structural variants were the only alterations in mesotheliomas; multiple alterations were common in cholangiocarcinoma and deep deletions were typically detected in ovarian serous cystadenocarcinoma, sarcomas and only in uveal melanoma.

**Fig. 3:**
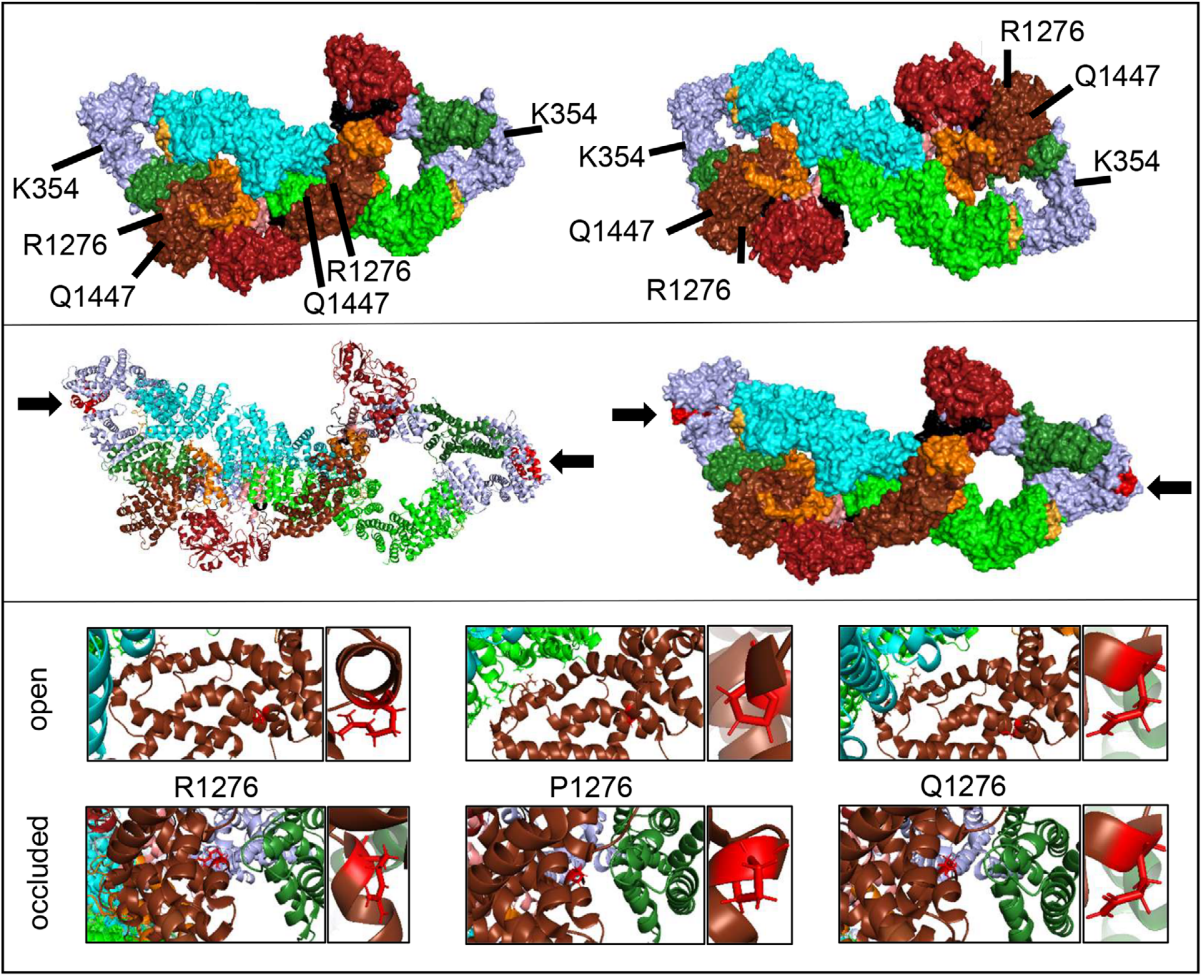
The neurofibromin isoform [i2839] structure derived from Naschberger et al. [31] (7PGT/U) with labelling of relevant regions in different colors for better distinction: N‐HEAT (light blue), CSRD (forest green), TBD (salmon), GRD (orange), RasGAP (chocolate), Sec14‐PH (fire brick), C‐HEAT (green/cyan), S‐rich domain (bright orange), linker 2 and 3 (black). We demonstrated localization of the residues prone to missense pathogenic variants Q1447H, R1276Q, and R1276P (GRD) or to aberrant splicing such as K354K (c.1062 G>A p.K354=), which is leading to deletion of exon 9 of C‐HEAT. The residues of the dimer are demonstrated in both protomers of open (upper left) and occluded (upper right) neurofibromin isoform [i2839] as a surface view. The missing exon 9 (variant K354=) is marked in red within the protein structure in the cartoon view (middle left) and the surface view (middle right) within N‐HEAT of the open neurofibromin dimer formation [i2839] (one protomer open and one occluded). A closer view of residue 1276 with respect to normal R1276 and mutant (P1276, Q1276) in the open (upper) and occluded formation (lower) is shown below (cartoon). Here, the specific residue is shown enlarged next to the cutout. Within the occluded conformation the CSRD (forest green) is very close to the RasGAP domain (chocolate) containing the R1276. In contrast, in the open formation the CSRD is far away from RasGAP exposed to bind Ras then.

As missense mutations point to the affection of indispensable protein functions, we demonstrate their localization within the open and closed neurofibromin structure and included the frequently occurring *NF1* variants such as c.3827G>C (R1276P) according to cBioPortal database data (Fig. 3). Pathogenic variant K354 = is a synonymous alteration/‘silent change’ in NF1 patients. It induces altered splicing and results in exon skipping but seems to preserve the integrity of the reading‐frame [14]. Therefore, this silent variant results in altered splicing and subsequent (in frame) exon skipping but not in premature stop and thus disables the neurofibromin function to create a phenotype.

To compare our findings of the various *NF1* variants with further, but smaller previous studies, we compared them with different entity‐specific studies providing detailed *NF1* variants mainly reporting analyses on melanoma and lung carcinoma [15, 16, 17]: Cohort sizes ranged from 161 [melanoma] [15] to 56 [lung cancer] [17], and to 16 *NF1* variants [melanoma] [16] as integrated in Table S1 for comparison. Pairwise overlap degree of sets of variants demonstrated only a small overlap between the Mainz and other cohorts (9/63 variants) as well as among the melanoma cohorts themselves (Fig. S2). The most common variants observed were primarily truncating alterations, consisting mainly of nonsense and frameshift mutations. Variants occurring in at least two cohorts were as follows: Q519*, G311*, L844F, P931L, Q1218*, Q1395*, Q347*, Q912*, R1276Q, R2450*, S1838C, and S2495F. Variants Q519*, P931L, Q1395*, R1276Q, and R2450* were shared with our Mainz cohort.

The recurrent variant Q519* affecting three of six melanoma cases, two cases with MPNST and one angiosarcoma case was also reported in the cBioportal cohort; however, it was not one of the most frequent mutations and observed in < 1% (4/581) melanoma samples only.

### Recurrent pathogenic missense hotspot variants and association to known germline variants

Comparing the abovementioned datasets with the Mainz patient pool, the following pathogenic *NF1* missense variants were recurrent: R1870Q/X1870_splice, c.3827G>A/R1276Q, c.1062G>A/p.K354=, and c.4341G>C/p.Q1447H (Table 1). As studies include a strongly different number of cases, it is amazing to recognize that concerning significant and recurrent missense hotspots, the arginine finger R1276 occurs in three studies and seems to remain a clue to explain increased tumor development as it is the most essential element of neurofibromin for Ras binding. The R1276 residue is located within the GTPase‐activating protein (GAP) domain of neurofibromin, which interacts with the Ras protein to promote its conversion from an active GTP‐bound form to an inactive GDP‐bound form. The R1276P and R1276Q mutations both involve substitutions of the arginine (the positively charged arginine finger) with either proline or glutamine (both of which are polar but lack the guanidino group of arginine), which leads to alterations in the GAP activity of neurofibromin (Fig. 3).

In the cohorts, we did not see that recurrent pathogenic variants occurred exclusively in a specific tumor type, although some showed some preference, for example, for the nonsense variant R2540* for uterine endometrial carcinoma and Q519* in melanoma.

Finally, all variants but three that were discovered in our cohort had been described as germline *NF1* mutations before the remaining three comprised two variants that have not been reported before and one variant that had been described as somatic in lung carcinoma (c.3658G>T, compare annotations).

## Discussion

Various somatic *NF1* variants were detected in our patient pool. Nevertheless, due to our sequencing design intronic *NF1* gene variants as well as larger deletions and insertions were not covered. Therefore, the spectrum of variants displays a selection and is probably larger than detected in our patient pool. Among all *NF1* variants detected, only 32% were pathogenic. To be safe and have unchallenged evidence for the variants being relevant for tumor formation we included only those into our further analysis although likely pathogenic variants might play a role too. Interestingly, nonsense mutations were most common in our study, which contrasts with variants reported to databases (cBioPortal) where missense variants (3%) are fewer, and truncating variants (78%) are much more common among all driver mutations. Germline *NF1* mutations are reported with about 28% of missense/nonsense mutations in the literature [8]. This indicates a shifted spectrum of relevant *NF1* variants to nonsense and truncating mutations in tumors without disease NF1. If this reflects a change of mutation type due to affected DNA repair mechanisms remains to be investigated. Other studies reported similar findings [9]. Preferred sites for specific tumor entities are not seen in the patient pools. As most variants are nonsense mutations, the specifically affected domain might not be of major importance because truncated neurofibromin might prevent the dimer and subsequent tetramer formation in general and hamper the neurofibromin function dramatically as introduced above.

Comparing data of our patient pool with database collections, the entities with *NF1* variant frequency of > 10% differed. Some tumor entities, which have been frequently reported in the past, are not represented at all in our Mainz oncology patient pool. This could be explained by the MTB cohort itself, which has a different spectrum of patients. We here summarize key features that explain differences in frequencies (1), hotspots (2) or (3) tumor entities of the Mainz patient pool to database cohort:

(1) The Mainz cohort may show higher frequencies of mutations that are ethnically or regionally specific, while cBioPortal could capture a broader range of mutations across different entities and geographic regions. For example, mutations such as R1276P/Q or R2450*, which are recurrent in certain settings, may be less common in cBioPortal if they are regionally enriched in the Mainz cohort or vice versa. (2) Certain hotspot mutations may appear more frequently in one cohort due to shared environmental factors or genetic ancestry. For example, if certain mutations are associated with specific ethnic groups within the Mainz cohort, they could be underrepresented in the global data available in cBioPortal, which might dilute these regional patterns. (3) cBioPortal includes a broader range of tumor types where *NF1* mutations are present, such as gliomas, MPNSTs, and optic pathway gliomas, with mutation distributions specific to each cancer type. In contrast, the Mainz cohort may have a different representation, potentially skewed toward a particular tumor or age group (e.g., pediatric gliomas or neurofibromas). As our approach to establish the Mainz’ cohort was not unbiased, several features might not be well comparable; nevertheless, recurrent variants are of major interest for a potential use as molecular markers in a diagnostic setting.

The pathogenic variant R1276P (NM_001042492.3) was first described in an NF1 family with three affected children [5] and is reported frequently [18]. One parent carrying the mutation died before the age of 30 due to an MPNST. In a functional study, we could show that R1276P neither impairs the protein structure nor binding to Ras substantially but disables the GAP activity by a 1000‐fold decrease dramatically [5]. The GAP‐related domain of neurofibromin (GRD) undergoes a large movement while changing from closed to open conformation to facilitate binding of Ras. Further studies uncovered the role of the arginine finger R1276 deeply (thus this variant became one of the best studied mutations in NF1 [18, 19, 20, 21, 22]) showing an association of R1276P to more severe NF1 phenotypes, an association to glioma, a high prevalence of cardiovascular abnormalities and Noonan‐like features, bone abnormalities, fewer cutaneous but more plexiform neurofibromas and a high prevalence of symptomatic spinal neurofibromas [23, 24] and moreover, convincingly demonstrated abolished activity without reducing the neurofibromin stability [2].

The proven ability of R1276P to completely abolish the GAP makes this mutation a very significant one, the effect of which may be much stronger than those variants leading to truncations. Proline substitution probably introduces structural constraints to disrupt the GRD conformation and not only impairs arginine finger function impairing Ras' GTP hydrolysis. Except for the impaired binding of ligands, proline in general severely disturbs the protein structure of α‐helices (‘helix breaker’); proline substitution can especially affect α‐helix motifs as described for *Presenilin 1* variant L232 (c.695 T>C) in early‐onset Alzheimer's disease [25]. Introduction of mutations to proline massively broke helices and changed the antitumor, antibacterial, and cytotoxic effects, and DNA‐binding activity of other genes [26, 27].

Variant R1276Q (NM_001042492.3) of our study affecting the same position, but if the abolishment of activity is the same as for R1276P remains unclear. The glutamine substitution could have a somewhat different effect. The glutamine side chain is polar but lacks the proper structure to fully replace the positively charged arginine. It may affect the RAS binding and GTP hydrolysis function of neurofibromin similarly, leading to RAS hyperactivation. This could also result in increased signaling through the MAPK and PI3K‐Akt pathways, both of which are involved in promoting cell growth and survival, making cells more prone to transformation and tumor development.

Finally, nearly all variants had been described as germline *NF1* mutations before. For this reason, we ask ourselves whether all variants that enter the databases as somatic are truly somatic. In our opinion, this has not been proven by any study with a high degree of certainty, especially as sequencing is often only performed on tumor material (which was also the case in our cohort). To rule out that a cancer patient carries a germline *NF1* mutation or *NF1* mosaic mutation seems very significant. Mosaic *NF1* mutations might occur in several scenarios such as segmental NF (incidence of about 1 in 75.000), cell‐ or tissue‐specific germline mosaicism up to clonal late postmitotic mosaicism reported in hematopoiesis [28, 29]. Strictly speaking, a detected variant cannot be categorized as a somatic variant if germline mosaicism is not excluded. Contamination of samples by normal tissue can complicate the interpretation when a simple Mendelian approach is not possible and emphasizes the need for investigation of the surrounding tissue besides tumors as a control (since data from blood might not be conclusive enough). Other groups also suggested that *NF1* pathogenic variants are much more common than thought. Those authors could nicely show that somatic mosaicism (both postzygotic and clonal hematopoiesis), incomplete penetrance, and missed diagnoses contribute to an increased prevalence of pathogenic *NF1* variants in a genotype‐first approach [28]. Interestingly, in a study investigating gliomas, not only a high percentage of gliomas was linked to *NF1* germline variants but also the occurrence of constitutional mosaicism due to post‐zygotic events was detected in some cases, additionally again also affecting the arginine finger R1276 [24]. Finally, already antique illustrations of NF1 demonstrate remarkable mosaicism depicted by ancient observers [30].

What are the strengths and limitations of our approach? Our study, which considers the presence or absence of NF1 clinically, provides further evidence that a relevant subset of pathogenic *NF1* variants in tumors that do not traditionally present with NF1 may have an underlying genetic *NF1* mosaicism. This strength of the work should lead to further approaches to clarify this interesting question. Limitations are clearly the limited number of cases, the unbiased approach and the mutation analysis, which did not capture large aberrations due to technical limitations and thus narrowed the range of variants captured.

## Conclusions

We were able to show that *NF1* variants are detected in many different tumor entities in a setting when disease NF is not diagnosed or obvious. The distribution of *NF1* variants in our cohort is only partially consistent with the available data from the cBioPortal reflecting a bias of patient selection and mutation detection. In the Mainz patient pool lung carcinoma, glioma, malignant melanoma, sarcoma, and gynecological cancer were most frequently altered by pathogenic *NF1* variants and affected more female patients. Nevertheless, only in malignant melanoma and glioma did the frequency of pathogenic *NF1* variants reach 10% and was much lower in other tumor entities. Retrieved data from cBioPortal detected more variants in melanoma, gynecological, lung cancer, urothelial carcinoma, and pheochromocytoma with the most common *NF1* variants R2540*/Q, R1362*, R440*/Q/P, Q519*, R1306Q/*, I697Dfs*21/P678Rfs10, R1870Q/X1870_splice, Q400*, and R1276Q. The smaller Mainz cohort demonstrated recurrent pathogenic variants such as Q519*, S1053*, and D2346Vfs*49, whereas R1240* and R1276Q were similarly detected. Somatic variants are more frequent of nonsense/truncating type than reported for germline. Some of the common missense variants in the GRD are particularly dependent on the conformational change and function as a Ras GTPase. As some residues show recurrent change in different datasets such as R1276 and R2450, they represent an important hotspot and can be recommended to serve as biomarkers, probably with R2450* for endometrial cancer when not to use *NF1* variant alterations as a predictive biomarker in general. The data is sufficient to give *NF1* analysis a fixed part of cancer panels in routine. We critically question whether all *NF1* variants that have been reported in tumors without the presence of the disease NF1 are only somatic in cancer cells. As blood is the most common sample used in genetic testing, it may not reflect the mosaicism present in specific tissues or tumors. Therefore, mosaic variants can be missed if not assessed in the tumor microenvironment or other relevant tissues. Standard sequencing technologies, particularly tumor‐only next‐generation sequencing (NGS), may struggle to identify low‐level mosaicism, especially when the mutant allele frequency is below the threshold of detection or if it is outcompeted by the wild‐type allele. We suggest that further studies should increasingly exclude *NF1* mosaics or investigate whether this hypothesis can be refuted. The particularly high mutation rate of the *NF1* gene tends to favor multiple manifestations of mutations, also in the form of multiple types of mosaicism. Current methodologies, including standard sequencing and tumor profiling, may not capture somatic mosaic mutations that play a significant role in tumorigenesis. Integrating mosaicism as a factor in diagnostic guidelines, and considering it in genetic counseling sessions, would aid in the early identification of individuals who may be at increased risk for NF1‐associated tumors, even in the absence of full‐blown NF1‐associated disease. Recognizing and addressing NF1 mosaicism in clinical practice requires multi‐tissue sampling, enhanced sequencing technologies, and a more inclusive approach to genetic testing.

## Conflict of interest

The authors declare no conflict of interest.

## Supporting information


**Table S1.** Overview of all detected NF1 variants in our institutional cohort with respect to gender, age, and tumor type.

**Fig. S1.** Overview of the Mainz Oncology center patient pool with somatic *NF1* gene variants.

**Fig. S2.** Heatmap (A) and Jaccard (B) indices of pairwise comparisons of NF1 variants from the Mainz cohort with previous studies.

## Data Availability

All variants of the study were submitted to ClinVar (https://www.ncbi.nlm.nih.gov/clinvar/). Other datasets analyzed are available from the corresponding author upon reasonable request.
