# Supplementary material for: Pathogenic Neurofibromatosis type 1 gene variants in tumors of non‐NF1 patients and role of R1276
Source: FEBS Open Bio. 2025 Nov 11;16(4):803–13. doi: 10.1002/2211-5463.70157 (PMC13042986; doi:10.1002/2211-5463.70157)
Supplement: Supplementary file 2 — Fig. S1. Overview of the Mainz Oncology center patient pool with somatic NF1 gene variants. [file FEB4-16-803-s003.pdf]

Figure S1

Overview of the Mainz Ocology centre patient pool with somatic *NF1* gene variants

A) Percentage distribution of different tumor entities demonstrating *NF1* variants irrespective to type of variant. The designation „Others“ includes breast carcinoma, urachus carcinoma, penis carcinoma, leukemia (AML), oropharynx carcinoma, renal cell carcinoma, thyroid carcinoma, neuroendrocrine tumor, and giant cell tumor.

B) Age range of cases with *NF1* variants. Young cases are itemized for tumor entity and type of variant.

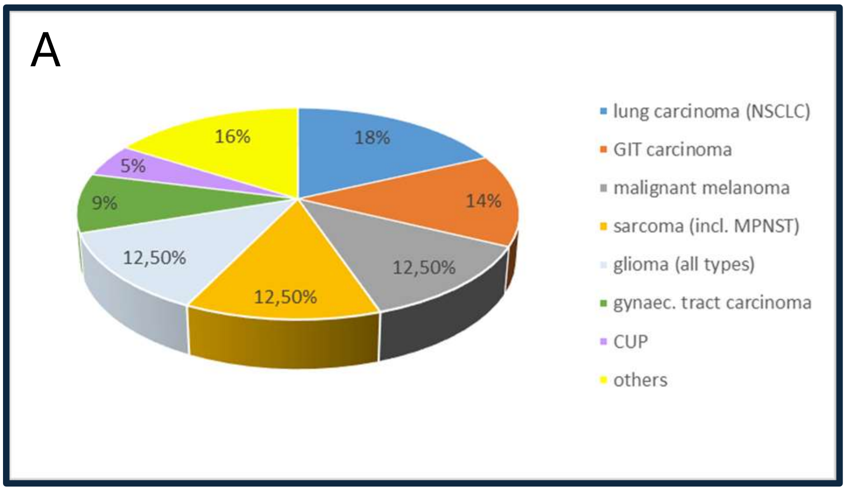

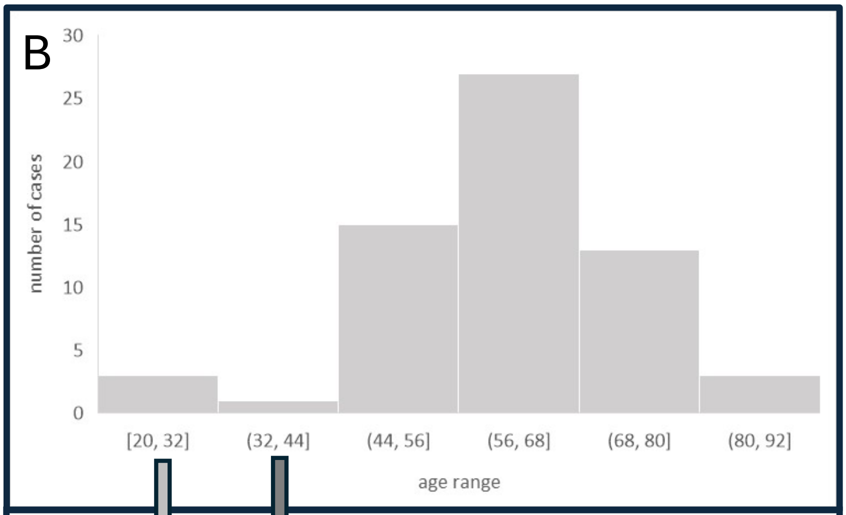

n = 3

n = 1, **colon sigmoideum carcinoma** (NF1 variant c.4541A>G, *NF1* variant of uncertain significance)

- **giant cell tumor** (*NF1* variant of uncertain significance: c.3038C>T; NM_001042492.3)
- **colon carcinoma** (two pathogenic *NF1* variants: c.5986del and c.2252-1G>T)
- **acute myeloblastic leukemia** (recurrent pathogenic *NF1* variant: c.2033dup; NM_001042492.3)
