# Supplementary material for: Pathogenic Neurofibromatosis type 1 gene variants in tumors of non‐NF1 patients and role of R1276
Source: FEBS Open Bio. 2025 Nov 11;16(4):803–13. doi: 10.1002/2211-5463.70157 (PMC13042986; doi:10.1002/2211-5463.70157)
Supplement: Supplementary file 3 — Fig. S2. Heatmap (A) and Jaccard (B) indices of pairwise comparisons of NF1 variants from the Mainz cohort with previous studies. [file FEB4-16-803-s001.pdf]

Figure S2

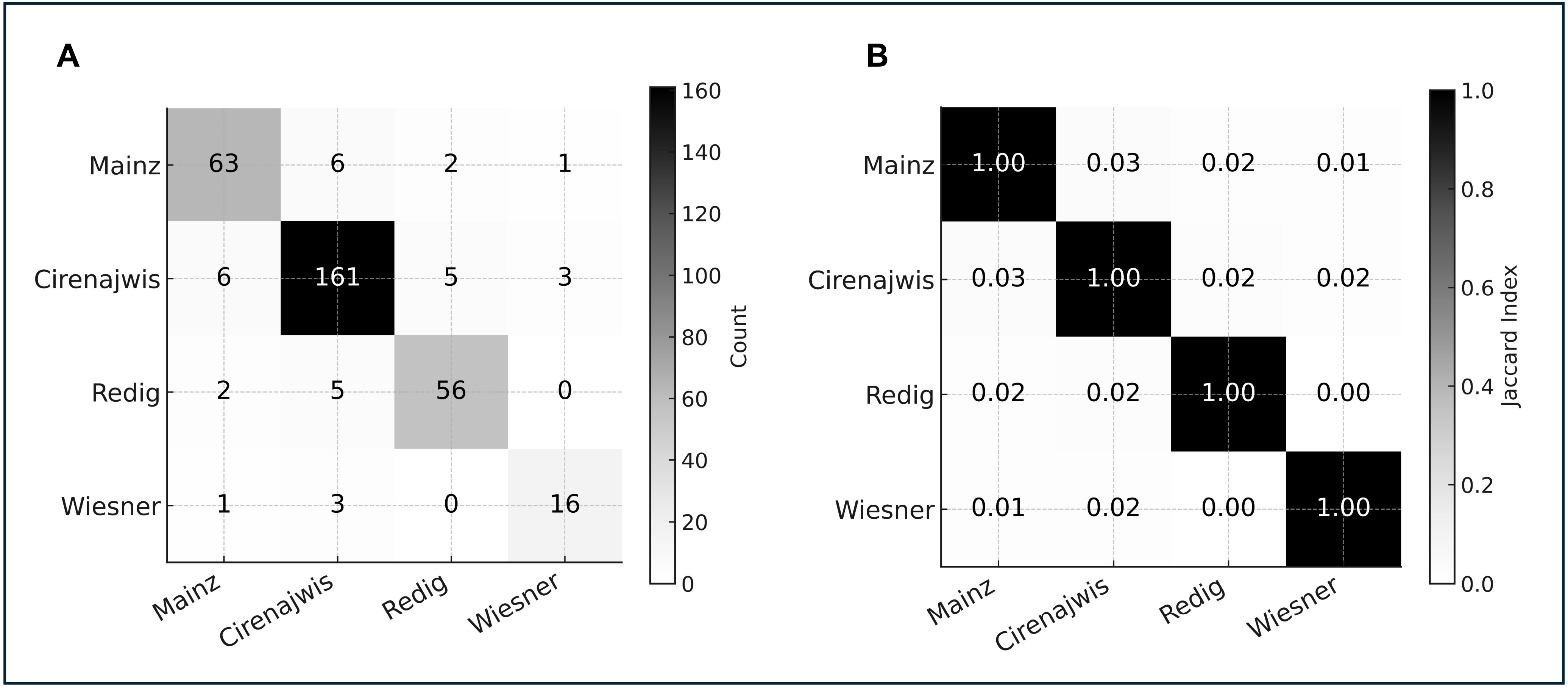

Heatmap (A) and Jaccard (B) indices of pairwise comparisons of NF1 variants (data from Mainz cohort and from studies of Cirenajwis et al., Redig et al., and Wiesner et al. (15-17).
